# Diagnostic value of 3D dynamic contrast-enhanced magnetic resonance imaging in lymph node metastases of head and neck tumors: a correlation study with histology

**DOI:** 10.1177/2058460120951966

**Published:** 2020-08-26

**Authors:** Christoph Treutlein, Adrian Stollberg, Claudia Scherl, Abbas Agaimy, Stephan Ellmann, Heinrich Iro, Michael Lell, Michael Uder, Tobias Bäuerle

**Affiliations:** 1 Department of Radiology, University Hospital Erlangen, Erlangen, Germany; 2 Department of Otorhinolaryngology, Head and Neck Surgery, University Hospital Erlangen, Erlangen, Germany; 3 Institute of Pathology, University Hospital Erlangen, Erlangen, Germany; 4 Department of Radiology and Nuclear Medicine, Klinikum Nuremberg, Paracelsus Medical University, Nuremberg, Germany

**Keywords:** Dynamic contrast-enhanced magnetic resonance imaging, head and neck neoplasms, lymph nodes, lymphatic metastasis, magnetic resonance imaging

## Abstract

**Background:**

Accurate staging of cervical lymph nodes (LN) is pivotal for further clinical management of patients with head and neck cancer. Functional magnetic resonance imaging (MRI) such as three-dimensional (3D) dynamic contrast-enhanced (DCE) acquisition might improve the diagnosis of cervical LN metastases.

**Purpose:**

To evaluate the additional diagnostic value of high-resolution 3D T1-weighted DCE in detecting LN metastasis compared to standard morphological imaging criteria in patients with head and neck tumors as correlated to histopathology.

**Material and Methods:**

Standard MRI with 3D DCE acquisition at voxel sizes of 1 × 1×1 mm was performed in 15 patients before surgery; 92 LN of the head and neck were histopathologically analyzed. A logistic regression analysis of semi-quantitative DCE parameters, time-intensity curve (TIC) shapes, and morphological criteria was performed to differentiate benign from malignant LN.

**Results:**

Standard MRI was sufficient for diagnosis of malignancy in LN with a short-axis diameter ≥ 15 mm (n = 17). For LN metastases with a short-axis diameter <15 mm (n = 12), however, the combination of 3D DCE MRI parameters, TIC shapes, and LN diameter significantly increased the sensitivity and specificity of diagnosing metastases (DCE + TIC shape + LN diameter: 92% and 88% vs. DCE only: 83% and 68% (*P* < 0.01) vs. LN diameter only: 83% and 77% (*P* = 0.04).

**Conclusion:**

MRI including isotropic high-resolution 3D DCE acquisition combined with morphological criteria allows an accurate assessment of small cervical LN metastases in patients with head and neck cancer. For LN ≥ 15 mm diameter, morphologic imaging may suffice to diagnose metastatic disease to the LN.

## Introduction

Head and neck cancer is the ninth most common tumor worldwide and >90% of these cancers are squamous cell carcinomas, originating from the epithelium of the mucosal lining (1). The five-year survival rate is around 60% (2). After local therapy, the development of cervical lymph node (LN) metastases is the most important factor influencing the patients’ prognosis (3,4). Both prognosis and therapy of the patients depend, in particular, on the extent of regional LN spread. Patients with LN metastases at levels 3 and 4 seem to have a greater risk of developing distant disease (5). To control disease above the level of the clavicles is crucial for a favorable outcome in patients with head and neck carcinoma. The treatment approach includes surgery, radiotherapy, chemotherapy, or a combination depending on the stage according to the American Joint Committee on Cancer (AJCC) TNM staging system (5). The mainstay of surgical management of metastatic neck cancer has been neck dissection (6). Thus, accurate staging of cervical LN is pivotal in the planning of the further clinical management, particularly to weigh the possible curative effect against the risk of morbidity or complications of neck dissection (7–10).

Current imaging techniques play an important role in the assessment of nodal metastases. Mainly, radiological diagnostic criteria for malignancy of LN are almost exclusively based on morphology and the presence of nodal hypoperfusion (11,12). However, the reliance on morphological and size-related criteria of computed tomography (CT), magnetic resonance imaging (MRI), and ultrasound bears some inherent disadvantages. Detection of small metastatic deposits and differentiation between inflammatory and metastatic enlarged LN may fail to provide an accurate assessment (13). In addition, fluorodeoxyglucose (FDG)-positron emission tomography (PET) is not always able to solve this problem due to low spatial resolution and the variable physiological uptake of FDG in inflammatory LN (14,15).

The primary morphological criteria for diagnosis of nodal metastases are diameter, radial length, eccentricity, rectangularity, surface, volume, and sphericity with moderate sensitivity and specificity values (11). Sensitivity and specificity of short-axis diameter, in particular, were in the range of 61%–92% in different studies (16). Threshold values with a maximum size in the range of 5–10 mm were proposed by different authors in order to differentiate benign from malignant nodes, while exceeding this threshold value LN nodes were considered metastatic (16).

Beyond morphological and size-related criteria, functional MRI techniques, such as dynamic contrast-enhanced (DCE) MRI as a quantitative technique to non-invasively assess the microvasculature within tissue (17), arterial spin labelling and diffusion MRI (18), MR spectroscopy (19), and dynamic susceptibility perfusion contrast MRI (20) are promising methods for the differentiation of benign and malignant LNs. In addition, CT-based functional imaging techniques such as perfusion CT may have a role in the detection of metastatic LN, according to Razek et al. (21).

However, still no routine imaging technique is available to reliably diagnose small metastatic LN in patients lacking obvious criteria of malignancy. Therefore, the accuracy of preoperative nodal status with current imaging techniques is still limited (12).

Clearly, histopathological methods are still routine in the staging and treatment planning of head and neck cancer (7,8). Selective neck dissection is considered the best option to confirm nodal status in these tumors, while preoperative sentinel node biopsy are still considered investigational, although it is recognized as a gold standard in other malignant entities such as melanoma or breast cancer (22).

In the present study, we performed isotropic high-resolution three-dimensional (3D) DCE MRI on a group of patients with malignant head and neck tumors before surgery. The aim of the present study was to evaluate the additional diagnostic value of adding 3D DCE MRI to morphological imaging for the assessment of LN metastases in patients with head and neck tumors as correlated to histopathology.

## Material and Methods

### Study design and patient characteristics

Written informed consent was obtained from each patient before MRI. In 2016, 15 patients with malignant head and neck tumors, histologically verified by fine needle biopsy or partial surgical excision, underwent DCE MRI scans before primary surgery with neck dissection. Neck dissection was performed as modified radical neck dissection or extended modified radical neck dissection depending on preoperative neck status (cN status) and intraoperative findings. Specimens were taken according to exact anatomical neck levels from 92 LN. Each neck level was marked and sent to histological examination separately. All surgically excised LN were histologically analyzed and correlated to MRI images. Patients were excluded for any of the following reasons: prior cancer diagnosis except that of appropriately treated localized epithelial skin cancer or cervical cancer; prior radiotherapy to the head and neck; and contraindications for gadolinium-based contrast agents.

### MRI examination

All MRI examinations of the neck and axilla were performed on a 3-T platform (Skyra; Siemens Healthcare, Erlangen, Germany). The MRI protocol was as follows:

- Coronal and axial turbo pre-contrast inversion recovery magnitude (TIRM) T2-weighted (T2W): TR = 2000 ms; TE = 32 ms; slice-selective IR with TI = 220 ms; flip angle = 150°; slice thickness = 4 mm; base resolution = 256; voxel size = 0.9 × 0.9 × 4.0 mm; parallel imaging GRAPPA = acceleration factor 2.
- CAIPIRINHA-Dixon-TWIST (CDT)-volume-interpolated breath-hold examination (VIBE): TR = 4.57 ms; TE1 = 1.51 ms; TE2 = 2.56 ms; flip angle = 12°; acquisition time = 29 s; measurements = 20; slice thickness = 1.0 mm; base resolution = 256; voxel size = 1.0 × 1.0 × 1.0 mm; parallel imaging CAIPIRINHA = acceleration factor 2; Gadobutrol (Gadovist, Bayer HealthCare Pharmaceuticals) was administered to the patients (dose, 0.1 mmol/kg) followed by a 20-mL saline flush via a power injector at a rate of 3 mL/s; field of view = 260 × 260 mm. Total scan time was 3 min 50 s.
- Coronal and axial post-contrast T1-weighted (T1W) turbo spin echo (TSE) with fat saturation: TR = 626 ms (axial 579 ms); TE = 9.2 ms (axial 9.9 ms); flip angle = 150°; slice thickness = 4.0 mm; fat suppression = SPectral Attenuated Inversion Recovery (SPAIR); base resolution = 320; voxel size = 0.8 × 0.8 × 4.0 mm; parallel imaging GRAPPA = acceleration factor 2.

### Image analysis

The histopathologically analyzed LN were identified on MRI sequences according to size and the anatomical location of levels (I–VII) (4) and were consensually reviewed by two radiologists (CT and TB) with four and 12 years of experience, respectively. The identified LN were measured in long and short axes.

**Fig. 1.**
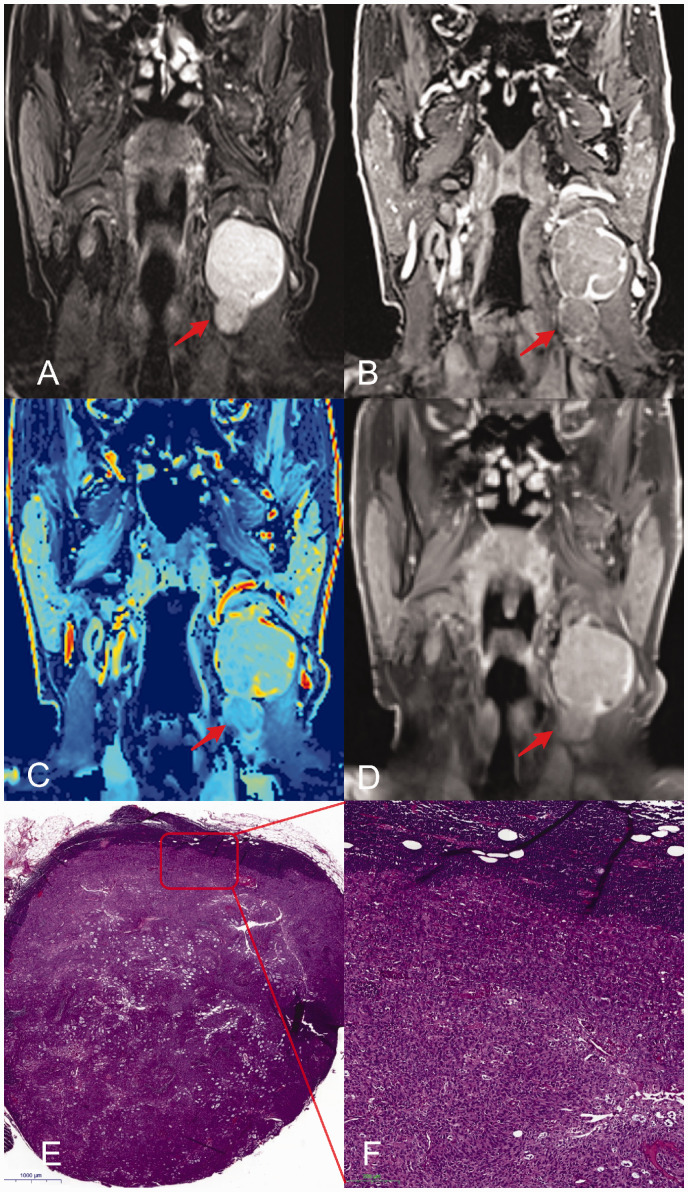
Representative images of a patient with squamous cell carcinoma and a metastatic cervical LN with a short-axis diameter of 18 mm. Metastatic LN (arrow) on coronal images from T2W TIRM (a), 3D DCE (b); 3D DCE derived pixel-by-pixel color-coded map of the iAUC (red = maximum, blue = minimum) (c); contrast-enhanced T1W imaging (d); histological analysis (e, H&E staining; overview); close-up of metastatic LN (f). DCE, dynamic contrast-enhanced; iAUC, initial area under the curve; LN, lymph node; T1W/T2W, T1-/T2-weighted.

Regions of interest (ROIs) were drawn manually by encircling the entire LN at an anatomical midline location (Syngo.via, Siemens Healthcare GmbH, VB 30A). For each ROI, perfusion variables were extracted as follows: average slope in the initial 30 s of measurement (wash in); average slope in the last 30 s of measurement (wash out); time to peak enhancement (TTP); peak enhancement (PE) ratio defined as (Smax – S0)/S0 (Smax – maximum signal intensity and S0 – pre-contrast signal intensity); and initial area under the curve (iAUC) defined for the first 60 s. Time-intensity curve (TIC) shapes were created for the different ROIs in DCE images and classified into six different groups: (Ia-c) slow uptake and (IIa-c) rapid uptake of contrast agent in the initial phase (Fig. 1). The delayed phase could be divided into a horizontal “plateau-phase” (±10%) and an increasing (>10%) and decreasing ( < 10%) phase. Intra- and inter-reader reliability of TIC shape assessment was performed after an interval of four weeks. LN with low attenuation centers with or without a surrounding zone of enhancement in DCE sequences and a corresponding blue to black coloring in DCE MRI maps were classified as hypoperfused.

### Histopathological correlation

All LN were histopathologically evaluated for evidence of tumor cells (metastatic LN). The pathologist was blinded to the MRI results. Every sample or LN was fixed with formalin and embedded in paraffin, followed by serial sectioning and permanent staining with hematoxylin and eosin.

### Statistical analysis

Assumptions of normality were checked by visual inspection of quantile–quantile plots with log-transformation. The Mann–Whitney U test was used for non-parametric comparisons. Agreement was assessed using weighted kappa statistics for ordinal measures. Multivariate logistic regression analyses with receiver operating characteristics (ROC) were performed with RStudio (RStudio Team (2015), Version 1.1.456: Integrated Development for R. RStudio, Inc., Boston, MA, USA; http://www.rstudio.com) and GraphPad Prism 6 (GraphPad Software, La Jolla, CA, USA). Confidence Intervals (CI) were set as 95% and level of significance was defined as *P* < 0.05.

## Results

The characteristics of patients with head and neck cancer and the respective locations of the primary tumors are shown in Table 1. One patient was excluded due to surgical complications and another patient due to an incomplete MRI examination (claustrophobia).

**Table 1.** Patients demographic data and tumor characteristics.

Baseline characteristics			
Patients (n = 13)	Age (years)	61.5 ± 6.7	
	Weight (kg)	78.2 ± 17.1	
	Height (cm)	173 ± 7	
	Contrast agent (mL; Gadovist®)	7.7 ± 1.8	
*Patient*	Primary tumor	Histopathological classification of lymph nodes	
		*Benign*	*Malignant*
1	Tongue carcinoma	3	2
2	Tongue carcinoma	5	3
3	Hypopharynx carcinoma	6	2
4	Larynx carcinoma	8	1
5	Tongue carcinoma	5	2
6	Larynx carcinoma	7	2
7	Hypopharynx carcinoma	5	2
8	Oropharynx carcinoma	13	4
9	Oropharynx carcinoma	2	5
10	Uvula carcinoma	1	0
11	Oropharynx carcinoma	6	5
12	Tongue carcinoma	0	1
13	Hypopharynx carcinoma	1	0
	Sum	62	29

Values are given as n or mean ± SD.

From 91 surgically excised and histopathologically analyzed LN in 13 patients (age = 61.5 ± 6.7 years, weight = 78.2 ± 17.1 kg, height = 173 ± 7 cm) with head and neck tumors, 29 were pathologically diagnosed as malignant and 62 as benign. All LN could be localized in all series of the MRI sequences with a median of seven per patient (range = 1–12). Out of 74 LN, 12 with a short-axis diameter <15 mm and all LN ≥ 15 mm (n = 17) were histopathologically classified as metastatic. Malignant LN presented with significantly larger long- and short-axis diameters and significantly lower iAUC, PE ratio, and wash-in rates in DCE MRI (Tables 2 and 3).

**Table 2.** Semi-quantitative 3D DCE values and morphological criteria of all evaluated LN.

	All lymph nodes		
	Benign (n = 62)	Metastatic (n = 29)	*P*
Wash in*	0.88 ± 0.34	0.55 ± 0.27	*<0.01*
Wash out^†^	–0.08 ± 0.34	0.01 ± 0.08	*<0.01*
TTP	86.50 ± 46.01	85.53 ± 42.57	*0.43*
PE ratio^‡^	0.78 ± 0.32	0.54 ± 0.36	*<0.01*
iAUC^§^	0.48 ± 0.18	0.29 ± 0.12	*<0.01*
Short-axis diameter	0.55 ± 0.27	2.02 ± 1.65	*<0.01*
Long-axis diameter	0.94 ± 0.45	2.72 ± 2.06	*<0.01*

Values are given as mean ± standard deviation.

*Average slope during initial (first 30 s) enhancement (mmol/min).

^†^Average slope during delayed (last 30 s) enhancement (mmol/min).

^‡^(S_max_–S_0_)/S_0_.

^§^First 60 s, a.u.min.

DCE, dynamic contrast-enhanced; iAUC, initial area under the curve; LN, lymph node; PE, peak enhancement; S_0_, pre-contrast signal intensity; S_max,_ maximum signal intensity; TTP, time to peak.

**Table 3.** Semi-quantitative 3D DCE values and morphological criteria of LN with short-axis diameter < 15 mm and ≥ 15 mm.

	Lymph nodes < 15 mm			Lymph nodes ≥ 15 mm
	Benign (n = 62)	Metastatic (n = 12)	*P*	Metastatic (n = 17)
Wash in*	0.87 ± 0.32	0.67 ± 0.19	*<0.05*	0.49 ± 0.25
Wash out^†^	–0.08 ± 0.46	–0.03 ± 0.10	*0.24*	0.02 ± 0.01
TTP	86.50 ± 45.91	94.50 ± 56.85	*0.46*	79.20 ± 26.69
PE ratio^‡^	0.78 ± 0.33	0.67 ± 0.43	*<0.01*	0.46 ± 0.23
iAUC^§^	0.48 ± 0.17	0.34 ± 0.07	*<0.01*	0.27 ± 0.12
Short-axis diameter	0.54 ± 0.22	0.98 ± 0.27	*<0.01*	2.56 ± 1.68
Long-axis diameter	0.94 ± 0.36	1.44 ± 0.39	*<0.01*	3.45 ± 2.17

Values are given as mean ± standard deviation.

*Average slope during initial (first 30 s) enhancement (mmol/min).

^†^Average slope during delayed (last 30 s) enhancement (mmol/min).

^‡^(S_max_–S_0_)/S_0_.

^§^First 60 s, a.u.min.

DCE, dynamic contrast-enhanced; iAUC, initial area under the curve; LN, lymph node; PE, peak enhancement; S_0_, pre-contrast signal intensity; S_max,_ maximum signal intensity; TTP, time to peak.

**Fig. 2.**
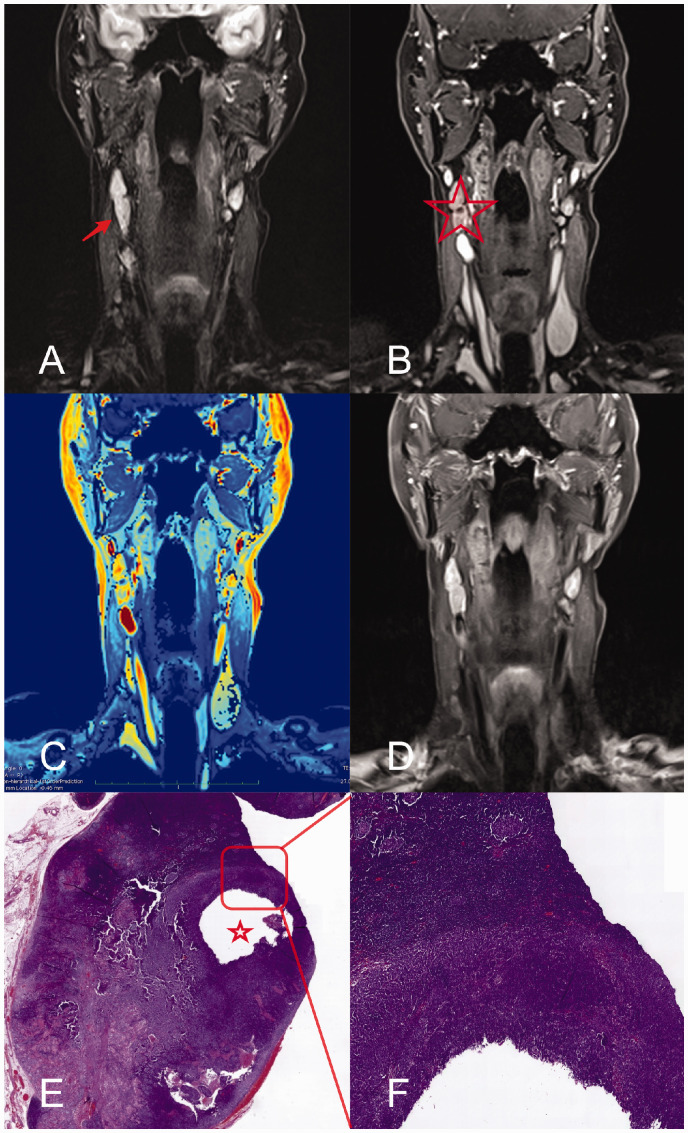
Representative images of a patient with non-keratinizing squamous cell carcinoma and a metastatic cervical LN with a short-axis diameter of 11 mm. Metastatic LN (arrow) on coronal images from T2W TIRM (a), 3D DCE (b); 3D DCE derived pixel-by-pixel color-coded map of the iAUC (red = maximum, blue = minimum) (c); contrast-enhanced T1W imaging (d); histological analysis (e, H&E staining; overview); close-up of metastatic LN (f). The hypoperfused area (asterisk) is delineable in the 3D DCE sequence only (asterisk) and confirmed histologically. DCE, dynamic contrast-enhanced; iAUC, initial area under the curve; LN, lymph node; T1W/T2W, T1-/T2-weighted.

**Fig. 3.**
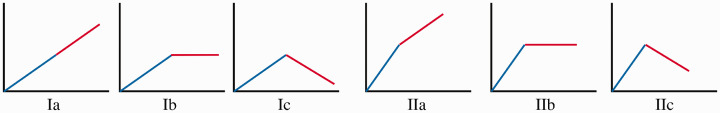
Classification of time-intensity curve shapes. Initial phase (black): (Ia–c) slow uptake; (IIa–c) rapid uptake of contrast agent. Delayed phase (green): (Ia and IIa) increasing (> 10%); (Ib and IIb) horizontal “plateau” phase (± 10%); (Ic and IIc) decreasing (> 10%).

All six TIC shapes occurred and could be differentiated. We observed TIC shape type IIc most frequently in benign LN (54%). In malignant LN, type Ia (28%) was more frequent than type IIc (24%). However, no significant differences were observed. Inter- and intrareader agreement of TIC shapes was excellent (w-kappa: 0.84, 95% CI = 0.72–0.95 and 0.97, 95% CI = 0.94–1.00). LN revealing hypoperfused areas (n = 23, 25%; 6 [7%] in lymph nodes with a diameter <15 mm, 17 [18%] in lymph nodes with a diameter ≥15 mm) were malignant as shown by histopathological analysis. Figs. 2 and 3 show representative examples of MR images of large (18 mm short axis, Fig. 2) and small (11 mm short axis, Fig. 3) LN compared to the respective histopathological sections. The hypoperfused nodal area in Fig. 3 is neither detectable in T2W nor in T1W post-contrast, but only on DCE images, and is considered malignant.

**Fig. 4.**
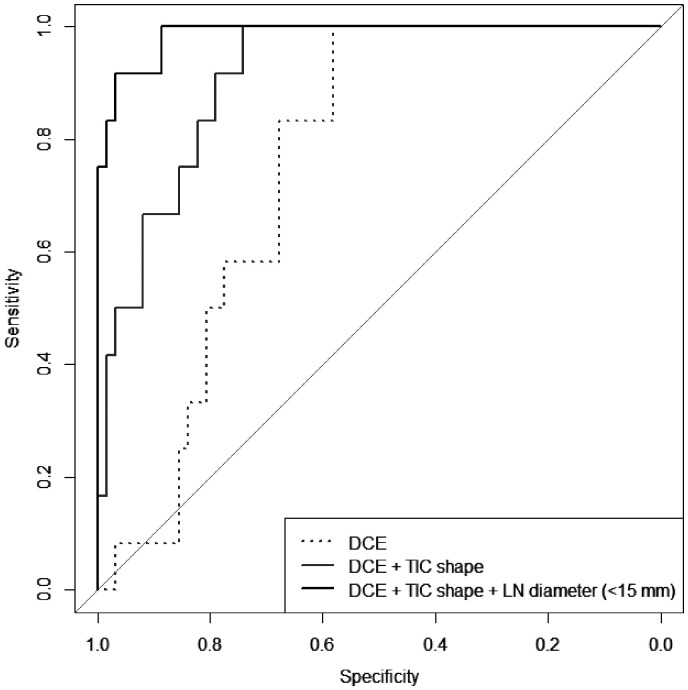
ROC curves depicting accuracy to discriminate malignant from benign LN: Dotted line: 3D DCE parameters (wash-in ratio, wash-out ratio, PE ratio, iAUC); Sensitivity 83%, Specificity 68%, AUC 0.76 (CI 0.67–0.86). Dashed line: 3D DCE parameters combined with TIC shapes; sensitivity = 83%, specificity = 84%, AUC = 0.91, 95% CI = 0.83–0.97; *P* = 0.02. Solid line: 3D DCE parameters combined with TIC shapes and LN diameter (<15 mm in short axis); sensitivity = 92%, specificity = 88%, AUC = 0.99, 95% CI = 0.95–1.00; *P* < 0.01. CI, confidence interval; DCE, dynamic contrast-enhanced; iAUC, initial area under the curve; LN, lymph node; PE, peak enhancement; ROC, receiver operating characteristic; T1W/T2W, T1-/T2-weighted; TIC, time-intensity curve.

ROC curves and respective AUCs (for LN < 15 mm) are depicted in Fig. 4. In small LN (short axis <15 mm) using generalized linear regression models, neither LN diameter nor DCE parameters nor TIC shapes were revealed as being statistically significant in terms of predicting malignancy. DCE parameters (wash in, wash out, PE ratio, iAUC, TTP; sensitivity = 83%, specificity = 68%) solely were not superior to LN diameter (sensitivity = 83%, specificity = 77%; *P* = 0.06) for predicting malignancy, but a combination of DCE parameters and TIC shapes could increase at least specificity from 68% to 84% (*P* = 0.02). A combination of DCE, TIC shapes, and LN diameter (<15 mm) increased sensitivity and specificity to 92% and 88%, respectively (*P* < 0.01; Fig. 4). The point estimates for AUC were largest for DCE at 0.76 (95% CI = 0.64–0.86), for LN diameter at 0.88 (95% CI = 0.77–0.97), for combined DCE and TIC shapes at 0.91 (95% CI = 0.84–0.97), and for combined DCE, TIC shapes, and LN diameter (<15 mm) at 0.99 (95% CI = 0.95–1.00).

## Discussion

The combination of semi-quantitative DCE parameters, TIC shapes, and LN size (short axis) significantly increased the diagnostic accuracy of diagnosing cervical LN metastases with a diameter <15 mm. In LN with a short-axis diameter >15 mm, there was no additional benefit of 3D DCE MRI parameters.

There are only few studies on the diagnostic ability of DCE MRI on metastatic LNs in head and neck tumors, primarily focusing on TIC shapes (23–25).

In contrast to Yan et al. (25), we found in the present study statistically lower perfusion values in DCE MRI of malignant LN, even in LN < 15 mm, most likely due to including the entire LN in the ROI for measurement without excluding nodal hypoperfused areas. However, our results with reduced wash-in and wash-out ratios and reduced PE in malignant LN are almost comparable to Fischbein et al. (24), except for the reduction of time to peak, which did not reach statistical significance in the present study. Thus, a decrease of overall perfusion in metastatic LN may result from partially hypoperfused areas. Interestingly, we found that areas of nodal hypoperfusion as detected by 3D DCE MRI was an important criterion to diagnose metastatic LN.

The diagnostic power of MRI in identifying metastatic cervical LN is limited, particularly with LN diameters <10 mm (26). According to RECIST 1.1, LN with a short-axis diameter >15 mm are considered potentially malignant, qualifying them as target lesions (27). In analogy to these criteria, the cut-off value of 15 mm in the short-axis diameter was chosen. Indeed, all LN with a diameter >15 mm were shown to be malignant. For the classification of these LN, we therefore do not see an advantage of performing additional DCE MRI sequences in an MRI standard protocol.

When focusing on LN < 15 mm, we describe the size of cervical LN as a significant variable for the assessment of LN metastasis, in contrast to Yan et al. (25) and Oztürk et al. (28). When considering the parameter “lymph node size” only, our results were within the range of previous studies: herein, we report a sensitivity of 83% and specificity of 77%, whereas other studies reported sensitivities in the range of 37%–90% and specificities of 71%–100% for conventional MRI (29). Van den Brekels et al. (30) stated that there was no imaging technique that reaches sensitivities > 75% without losing high specificity because of micrometastases that remain occult in all radiological imaging techniques. Importantly, the combination of semi-quantitative DCE parameters (wash in, wash out, PE ratio, TTP, iAUC), TIC shapes, and LN size significantly increased the sensitivity to 92% and specificity to 88% in our study. Compared to the present study, Razek et al. (20) reported for lymph nodes with a slightly higher sensitivity (97%) and lower specificity (83%), discriminating benign from malignant lymph nodes using the dynamic susceptibility-weighted contrast-enhanced perfusion MRI technique using 10 mm as the threshold for cervical LN.

For the assessment of small cervical LN, an optimized DCE MRI sequence is needed with high temporal and spatial resolution. Therefore, we have applied a CAIPIRINHA-Dixon-TWIST sequence at a 3-T MRI with an isotropic voxel size of 1.0 × 1.0 × 1.0 mm enabling 3D imaging of the volume of interest. Compared with the most recent publication on DCE MRI of cervical lymph nodes at 3 T using the TWIST technique, slice thickness was 3.0 mm resulting in increased voxel sizes compared to our sequence (25). A drawback resulting from the high isotropic resolution of 1 mm used in the present study is the increased scanning time for acquisition of a 3D dataset, resulting in potential miscalculation of the wash-in rate in the signal intensity versus time curve.

In the present study, semi-quantitative and heuristic DCE analyses were used instead of a pharmacokinetic DCE model, because these methods are simple to implement and robust in their performance, which facilitates their implementation in a clinical setting (31). As we assume a diagnostic benefit of adding a DCE sequence to head and neck MRI protocols from our results, we aim to perform further investigations in prospective clinical studies.

An important limitation of DCE MRI protocols is their lack of standardization (e.g. concerning contrast agent injection, spatial/temporal resolution, and post-processing algorithms). Thus, results are often of limited comparability between the centers, and institutions with protocols different from the ones used in the present study should handle the presented results with care. Another limitation is the study’s small sample size with many outcome variables, that may lead to reduced generalizability due to overfitting of the combined model (Fig. 4).

In conclusion, MRI combining isotropic high-resolution 3D DCE sequence parameters with morphological criteria allows an accurate assessment of small cervical LN metastases in patients with head and neck cancer. For LN with a short-axis diameter > 15 mm, morphologic imaging may suffice to diagnose metastatic disease to the lymph nodes.

## Supplemental Material

sj-pdf-1-arr-10.1177_2058460120951966 - Supplemental material for Diagnostic value of 3D dynamic contrast-enhanced magnetic resonance imaging in lymph node metastases of head and neck tumors: a correlation study with histology Click here for additional data file. Supplemental material, sj-pdf-1-arr-10.1177_2058460120951966 for Diagnostic value of 3D dynamic contrast-enhanced magnetic resonance imaging in lymph node metastases of head and neck tumors: a correlation study with histology by Christoph Treutlein, Adrian Stollberg, Claudia Scherl, Abbas Agaimy, Stephan Ellmann, Heinrich Iro, Michael Lell, Michael Uder and Tobias Bäuerle in Acta Radiologica Open
