# Supplementary material for: Diagnostic value of 3D dynamic contrast-enhanced magnetic resonance imaging in lymph node metastases of head and neck tumors: a correlation study with histology
Source: Acta Radiol Open. 2020 Aug 26;9(8):2058460120951966. doi: 10.1177/2058460120951966 (PMC7453466; doi:10.1177/2058460120951966)
Supplement: sj-pdf-1-arr-10.1177_2058460120951966 - Supplemental material for Diagnostic value of 3D dynamic contrast-enhanced magnetic resonance imaging in lymph node metastases of head and neck tumors: a correlation study with histology [file sj-pdf-1-arr-10.1177_2058460120951966.pdf]

Supplementary Table 1: Generalized linear regression models (GLM) predicting malignancy:

GLM	COEFFICIENTS	ESTIMATE STD.	ERROR	Z VALUE	PR(>|Z|)
**DCE + TIC SHAPE + LN DIAMETER**	Intercept	-2.028	5.534	-0.366	0.7141
	TIC 1b	2.749	6.747	0.407	0.6837
	TIC 1c	32.883	9.526	0.003	0.9972
	TIC 2a	-20.427	4.119	-0.005	0.9960
	TIC 2b	-8.078	5.444	-1.484	0.1378
	TIC 2c	1.156	3.024	0.382	0.7021
	iAUC	-13.517	12.106	-1.117	0.2642
	PE	9.175	7.539	1.217	0.2236
	TTP	-10.750	9.017	-1.192	0.2332
	Wash in	-5.390	9.514	-0.567	0.5710
	Wash out	-2.704	10.152	-0.266	0.7900
	LN diameter	16.474	8.665	1.901	0.0573
**DCE+ TIC SHAPE**	intercept	36.961	34.228	1.080	0.2802
	TIC 1b	-0.3522	25.334	-0.139	0.8894
	TIC 1c	249.044	69.604	0.004	0.9971
	TIC 2a	-204.198	26.968	-0.008	0.9940
	TIC 2b	-10.885	24.301	-0.448	0.6542
	TIC 2c	0.5957	27.652	0.215	0.8294
	iAUC	-181.207	103.266	-1.755	0.0793
	PE	41.171	43.057	0.956	0.3390
	TTP	-29.723	32.152	-0.924	0.3553
	Wash in	21.865	55.519	0.394	0.6937
	Wash out	109.664	177.343	0.618	0.5363
**LN DIAMETER**	intercept	-6.079	1.350	-4.503	0.000067
	LN diameter	5.934	1.540	3.853	0.000117
**DCE**	intercept	0.07449	183.426	0.041	0.9676
	iAUC	-1.435.202	750.790	-1.912	0.0559

	PE	0.10482	227.819	0.046	0.9633
	TTP	0.47568	164.069	0.290	0.7719
	Wash in	466.778	407.561	1.145	0.2521
	Wash out	117.791	137.610	0.856	0.3920
